# ICDs at higher age and clinical risk factors

**DOI:** 10.1007/s12471-014-0553-9

**Published:** 2014-05-03

**Authors:** W. Anné, D. A. M. J. Theuns, B. Schaer, Y. Van Belle, T. Szili-Torok, T. Smith, J. Res, L. Jordaens

**Affiliations:** 1 Department of Cardiology, Thoraxcenter–Ba 581, Erasmus MC, ’s Gravendijkwal 230, 3015-CE Rotterdam, the Netherlands; 2 Department of Cardiology, University Hospital of Basel, Basel, Switzerland

**Keywords:** Implantable cardioverter defibrillator, Survival, Age, Comorbidity, Geriatric cardiology

## Abstract

**Background:**

The implantable cardioverter defibrillator (ICD) is effective in preventing sudden cardiac death. However, in elderly patients (aged 75 years or older) the role of ICDs is still not well-defined and controversial.

**Methods:**

We retrospectively analysed all clinical and survival data of all ICD patients who were ≥75 years at the date of implantation in the Erasmus MC, Rotterdam, the Netherlands and the University Hospital, Basel, Switzerland. Kaplan-Meier survival analysis was performed, and mortality predictors were identified. Mortality of the cohort was compared with a random sample of patients aged 60–70 years originating from the same database and to an age- and sex-matched cohort of Dutch persons.

**Results:**

The study cohort consisted of 179 patients aged 75 years or older who were implanted between February 1999 and July 2008. The median follow-up time was 2.0 (IQR 2.8) years. Survival rates after 1, 2 and 3 years were 87, 82, 75 %, respectively. Survival was similar for primary and secondary prevention. Mortality in this study population could be predicted by combining four clinical risk factors: QRS duration >120 ms, NYHA class > II, renal failure and atrial fibrillation (AF). Survival was worse compared with the group of ICD patients aged 60–70 years and to the age- and sex-matched group of elderly persons. However, survival was not significantly worse when comparing elderly ICD patients without additional risk factors to the general population.

**Conclusions:**

Elderly patients still have an acceptable survival probability independent of prevention indication, certainly if there are no additional clinical risk factors. The presence or absence of additional clinical risk factors should be taken into account when making the decision for implantation, since they strongly correlate with survival.

## Introduction

Both in primary prevention and secondary prevention trials, there has been solid evidence that the implantable cardioverter defibrillator (ICD) reduces the rate of sudden cardiac death as well as the rate of total death [1–6]. Subanalysis of these large trials focusing on elderly patients showed that patients ≥75 years also profit from this therapy [7, 8]. However, due to budgetary restrictions in various countries, it is sometimes impossible to implant all ICD-eligible patients [9].

A risk stratification system was developed by Goldenberg et al. based on the MADIT 2 study [10]. The assessment of benefit of an ICD was based on a simple clinical score system including age, renal function, atrial fibrillation (AF), New York Heart Association (NYHA) class and QRS duration. Maximum benefit from an ICD was seen in patients with 1 or 2 risk factors, while no additional effect on mortality was seen in patients who had none or ≥3 risk factors. Furthermore, patients with severe renal failure as the only risk factor showed no benefit. We published similar observations in a group with heart failure treated with ICDs combined with cardiac resynchronisation therapy (CRT) [11].

In this retrospective study, we wanted to assess the survival of patients with ICDs (with or without CRT) who are 75 years or older and to study the impact of several risk factors on survival, using the MADIT 2 risk score as proposed by Goldenberg et al. as starting point.

## Methods

### Patient characteristics

A total of 179 consecutive patients over the age of 75 years and living in the region of Rotterdam, the Netherlands and the region of Basel, Switzerland, who received an ICD implantation, were included in this study. All ICDs were implanted between February 1999 and July 2008. They were followed up every 3 months at the ICD outpatient department and all data were carefully collected in the database each time. Clinical characteristics of the patients were analysed, and correlated with survival and with appropriate therapy. Mortality of the cohort was then compared with a random sample of patients aged 60–70 years originating from the same database, and with an age- and sex-matched cohort of the Dutch population obtained from the Dutch Institute of Statistics (www.cbs.nl) [12]. On this website survival data are given separately for men and women of every age. We constructed the survival curve for an age- and sex-matched cohort from these data, which is comparable with our study group.

Mortality was also determined using the modified risk factor model proposed by Goldenberg et al. (MADIT 2 risk score). The modification was necessary as obviously one of the risk factors, age ≥75 years, was an inclusion criterion on this study.

### Comorbidity

Comorbidity present before ICD implantation was identified through detailed analysis of the patient chart and laboratory data. Renal function was assessed by estimating the baseline glomerular filtration rate (eGFR) using the abbreviated Modification of Diet in Renal Disease (MDRD) Study equation: eGFR (mL/min/1.73 m^2^ of body surface area) = 186 × (serum creatinine in mg/dL)^−1.154^ × (age)^−0.203^ × 0.742 in female subjects. Impaired renal function was defined as an eGFR <60 mL/min/1.73 m^2^. Atrial fibrillation (AF) was considered to be present if there was at least one documented episode. Other reported comorbidity included cerebrovascular disease, chronic obstructive pulmonary disease, diabetes, peripheral vascular disease, renal failure, body mass index (BMI), and any malignancy (excluding metastatic cancer).

### Statistical analysis

Summary values are given as median and interquartile range (IQR). Categorical data are summarised as frequency and were compared using chi-square tests. Survival curves were composed using Kaplan-Meier analysis, and were compared by use of the log-rank test. Patients who were lost to follow-up were censored in the analysis from the time point of the last visit. The median survival was defined as the time point where the probability of survival is 0.50. A *p*-value of < 0.05 was considered statistically significant. The Cox proportional Hazard method was used to calculate hazard ratios of survival.

## Results

### Patient characteristics

Patient characteristics are summarised in Table 1.

**Table 1:** Patient characteristics

Age (years)	77 (IQR 3)
Male/female (%)	86/14
Device	
Single chamber device	40 %
Dual chamber device	35 %
Cardiac resynchronisation therapy	25 %
Indication	
Primary prevention	30 %
- Low ejection fraction (<35 %)	64 %
- Low ejection fraction (<35 %) + nsVT	36 %
Secondary prevention	
- Ventricular fibrillation	25 %
- Monomorphic VT	66 %
- Syncope suspected to be caused by VT	9 %
Ejection fraction	31 ± 9 %
<25 %	20 %
25–35 %	58 %
35–50 %	18 %
>50 %	4 %
Aetiology	
Ischaemic heart disease	82 %
Dilated cardiomyopathy	16 %
Miscellaneous	2 %
- Idiopathic ventricular fibrillation	2 patients
- Hypertrophic cardiomyopathy	1 patient
Functional class	
NYHA I	11 %
NYHA II	67 %
NYHA III	22 %
NYHA IV	0 %
Co-morbidity	
Moderate to severe renal failure	49 %
Diabetes	20 %
Atrial fibrillation	27 %
Peripheral arterial disease or stroke	24 %
COPD	13 %
History of malignancy	10 %
MADIT 2 classification	
0 points	0
1 point (age)	17 %
2 points	37 %
≥3 points	46 %
Medication use	
Beta-blockers	68 %
ACE-inhibitors/angiotensin II receptor blockers	71 %
Diuretics	75 %
Statins	66 %
Amiodarone	35 %
Digoxin	12 %

### Risk factors for mortality

**Fig. 1:**
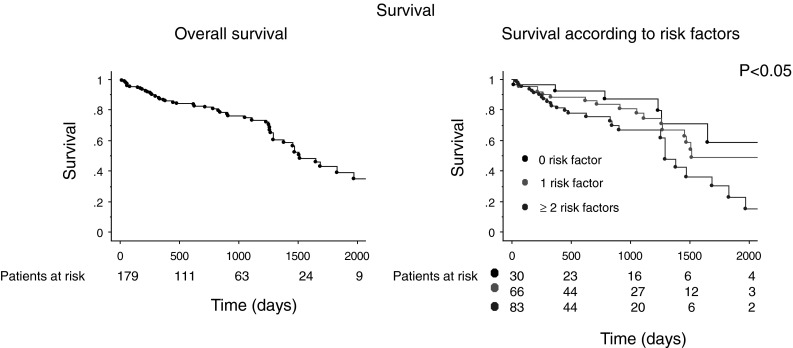
Kaplan-Meier curves of the overall survival in elderly patients (**a**) and the survival according to the different risk factors except age (**b**)

The median follow-up time was 2.0 (IQR 2.8) years. In total, 32 % of the patients died during follow-up after a mean of 74 months, 12 % of the patients were transferred to another hospital and lost for follow-up, and 56 % of the patients are still being actively followed. Survival after 1 year was 87 %, after 2 years 82 %, and 75 % after 3 years, respectively (Fig. 1a). Median survival was 4.2 years (IQR 2.4) years.

Risk stratification according to the four pre-mentioned risk factors (renal function, QRS duration, NYHA class > II, AF) showed a good survival in patients with 0 or 1 additional risk factors (except for age): 1-year survival was 93 and 88 %, respectively, 2-year survival 93 and 84 %, respectively. On the other hand, patients with ≥2 additional risk factors had a 1-year survival of 83 % and a 2-year survival of 76 % (*p* = 0.02) (Fig. 1b). Median survival was 6.2 (IQR 5.9) years in the patients with no additional risk factors (except of age), 4.2 (IQR 6.5) years in patients with one additional risk factor and 3.5 (IQR 3.2) years in patients with ≥2 additional risk factors. In the group of patients with no additional risk factors, 23 % died during follow-up, while this was 30 and 36 % for patients with 1 and 2 or more additional risk factors, respectively.

Age at the day of implantation, the reason for implantation (primary versus secondary prevention) ejection fraction, diabetes, body mass index, a history of myocardial infarction, the number of leads (VVI, DDD, CRT-D), the presence of peripheral vascular disease and pulmonary disease were not associated with an increased mortality. Only the need for diuretics was associated with a worse outcome.

Mode of death was cardiac in 42 %, with the majority of these due to heart failure (62 %). Non-cardiac death was recorded in 18 % while in 40 % the cause of death was not specified in the database.

### Risk factors for ICD therapy

**Fig. 2:**
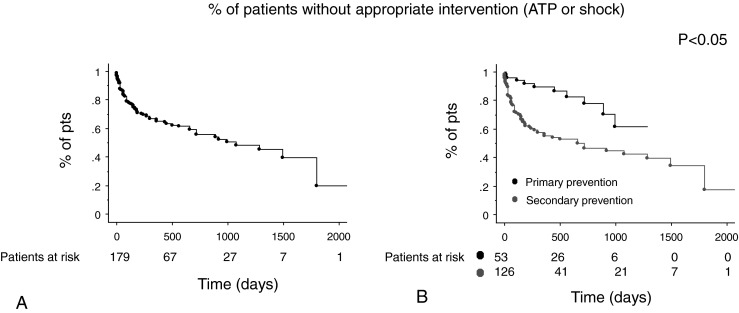
Kaplan-Meier curves of the percentage of patients receiving appropriate therapy in function of time as a whole (**a**) and according to the ICD indication (primary versus secondary) (**b**)

Of the patients, 41 % received an appropriate intervention of their device: 19 % of the primary prevention patients versus 50 % of the patients with secondary prevention indication (Fig. 2a and b). There was no difference in the proportion of patients receiving ICD therapies according to the MADIT 2 risk score: patients without risk factors (except for age) had a 50 % chance of an appropriate ICD therapy, those with 1 risk factor 42 % and those with ≥2 risk factors 36 % respectively (*p* = 0.39).

Clinical variables as age, NYHA class, AF, renal failure, medication use, history of myocardial infarction, and BMI were not associated with the chance of receiving an appropriate ICD therapy. Remarkably, the probability of receiving therapy was lower in our diabetic population (*p* = 0.03).

**Fig. 3:**
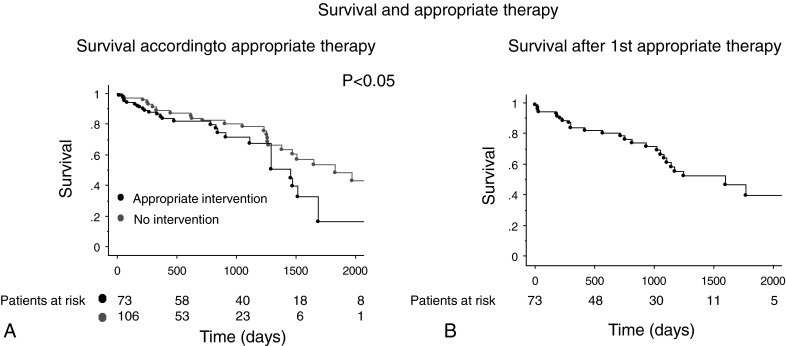
Kaplan-Meier curves of survival according to appropriate therapy or not (**a**). Kaplan-Meier curve of the survival after an appropriate shock

The median time to the first intervention was 101 (IQR 270) days. Mortality was higher among patients who experienced ICD therapy compared with those who did not (51 % vs. 30 %, *p* = 0.05) (Fig. 3a). The 1-, 2- and 3-year survival after an appropriate therapy was 84, 78 and 61 %. Median survival was 4.4 (IQR 5.9) years after an appropriate therapy (Fig. 3b). In every group of patients (according to their additional risk factors) the risk of dying was higher when appropriate therapy was needed.

Forty-nine percent of the patients who died, died without any ICD intervention (28 out of 57 patients). In patients with 0 or 1 additional clinical risk factors, 29 % died without any intervention (7 out of 24) versus 64 % in the group of patients with ≥2 additional clinical risk factors (21 out of 33) (*p* = 0.01).

### Comparison of survival

The distribution of ventricular (VVI), atrioventricular (DDD) and biventricular ICDs in the younger population was comparable with the group of elderly patients (not significant).

**Fig. 4:**
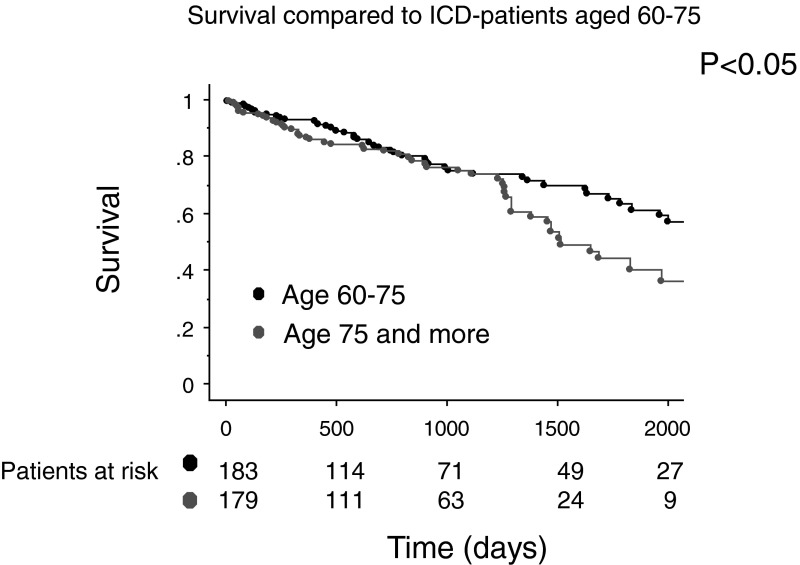
Comparison of survival between elderly ICD patients and a control group (60–70 years). The survival curves only started to separate after 3 years of follow-up

Survival was significantly better in the control group of ICD patients aged 60–70 years compared with the study group (*p* = 0.02). In the younger group of patients, 1-, 2- and 3-year survival was 93, 83 and 75 %, respectively, versus 87, 82 and 75 %. The survival curves only start to separate after 3 years of follow-up (Fig. 4). The median survival rate cannot be calculated since the time point that the probability of survival is 0.50 was not reached.

**Fig. 5:**
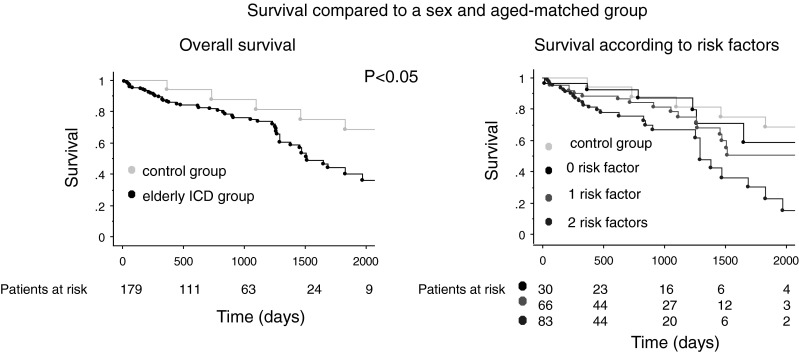
Comparison of the survival between the general elderly ICD patients (**a**) and according to the risk factors (**b**) and a sex- and aged-matched control group of the general population

Survival was significantly better in a sex- and age-matched group of the general (elderly) population of inhabitants of the Netherlands. Survival after 1, 2 and 3 years in this sex- and age-matched group is 94, 88 and 81 %, respectively, as compared with 87, 82 and 75 % in the study population (Fig. 5a). In the group of patients with no additional clinical risk factors (MADIT 2 risk score 1,–age), there was no statistical difference in survival between the general elderly Dutch population and our patients (*p* = 0.65). Survival in the latter group was 93, 93 and 88 % after 1, 2 and 3 years of follow-up (Fig. 5b). Compared with the general population the hazard ratio for death was 1.2 [0.5–2.7] for the patients with no additional risk factor (*p* = 0.66); 2.0 [1.2–3.4] for 1 additional risk factor and 3.6 [2.2–5.6] with ≥2 additional risk factors, except age.

## Discussion

The main findings of this study are that survival in elderly patients who need an ICD is good, certainly in those without additional risk factors. When no additional clinical risk factors were present, survival was even comparable with the general elderly population in the Netherlands. Survival was, as expected, worse in the elderly compared with a group of younger ICD patients, but the curves only started to diverge after 3 years of follow-up.

### Risk stratification

Risk stratification is an important tool, which can guide us to predict which patients have most benefit from the implantation of an ICD. Different strategies have been proposed such as T-wave alternans [13, 14], signal-averaged electrocardiography [15, 16], invasive electrophysiological study [17] and heart rate variability [16]. The risk stratification first described by Goldenberg et al. is simple and based on clinical risk factors [10]. Applied to the primary prevention MADIT 2 trial it proved to be an effective tool to help in distinguishing those who will benefit from an ICD from those who are too well or too sick to gain benefit from an ICD. When translating this risk scheme to our mixed primary and secondary prevention group of elderly patients, it proved to be a good predictor of mortality as well. Some of these risk factors (QRS duration, age, renal function) were also seen in a recent paper looking at outcome in a cohort of Dutch ICD patients [18].

Due to the increasing age of the population in Europe, the group of elderly patients with primary and secondary prevention indications will increase in number. Our data implicate that age in itself should not solely be a reason for exclusion from ICD implantation. Patients without any additional risk factors except age (MADIT 2 risk score: 1) had a survival comparable with the general elderly population. At first sight this may seem contradictory since we studied a group of patients with severe cardiac disease. However, when assessing the survival curve of a general age-matched elderly population, this survival curve includes not only healthy elderly people but also persons with different pathology (e.g. cancer). Furthermore, the survival curves between the younger ICD patients (60–70 years) and our elderly group of patients only started to diverge after 3 years of follow-up.

Implanting an ICD in a patient is not only based on scientific data but also on budgetary reasons in some countries, mostly in agreement with the guidelines [19]. Simple risk stratification with easily obtained clinical factors such as renal function, QRS duration, NYHA class and AF, can be a guidance in the process of decision-making, certainly in the elderly.

We do not say that ICDs are not useful in elderly persons with ≥2 additional clinical risk factors since this is a retrospective study not randomising patients. Survival after ICD and benefit from ICD are separate issues. However, patients with ≥2 additional clinical risk factors die sooner and with a higher chance of not receiving appropriate therapy, compared with those with 0 or 1 additional risk factor.

### Comparison with other studies

In a sub-analysis of the MADIT 2 trial, it was found that implanting an ICD in patients ≥75 years was associated with a similar risk reduction in total mortality compared with younger patients (44 % versus 37 %), implicating that age in itself might not be an exclusion criterion for ICDs [7]. Also, Koplan et al. found that in octogenarians with an ICD the median survival is 4.2 years, which matches well with our data [8].

The implantation of a transvenous ICD is no longer a major procedure and not associated with a long convalescence. The risks associated with implantation are minor and there is no decrease in quality of life after an ICD implantation [20].

This study only addresses mortality and not quality of life or cost-benefit. These factors are of equal importance in an elderly group of patients. According to recent studies costs remained under 100,000 $ per quality adjusted life year gained when the expected survival was ≥7 years [9, 21]. Median survival in our group is estimated at around 4.2 years implicating that the cost per quality adjusted life year gained is higher. However, when we only assessed patients with no additional clinical risk factors except age the median survival became 6.2 years. Furthermore, in recent years costs of ICDs have decreased in Europe which will probably lead to a lower cost per quality adjusted life year [9].

## Limitations

This study is descriptive and retrospective in nature and is not a randomisation of elderly patients to ICD implantation or optimal medical therapy. It only gives us an idea about survival in elderly patients in whom an ICD was already implanted. Therefore we do not have a control group of elderly non-ICD patients with similar characteristics. A selection bias is evident. All patients with the same risk profile as discussed in this study received an ICD. If an elderly ICD candidate did not receive the device this was probably due to a too high burden of comorbidity based on our clinical judgment. This may explain why the survival curves are similar between our younger and elderly ICD patients during the first 3 years. On the other hand, in younger patients selection bias will probably play a minor role compared with the elderly. Therefore, this study cannot be extrapolated to an unselected group of elderly patients needing an ICD for primary or secondary reasons.

The differences in the follow-up time between the primary and secondary prevention indications are due to the fact that we only started to implant for primary prevention reasons after the publication of MADIT 2 [1].

## Conclusions

Elderly patients, as selected in our hospitals, still have an acceptable survival, independent of prevention indication and therefore can still benefit from ICD therapy. It would be improper to withhold ICD therapy only based on age criteria, certainly if there are no additional clinical risk factors. The presence or absence of additional clinical risk factors should be taken into account when making the decision for implantation.
